# Prevalence of hypertension and prehypertension among children and adolescents in a semi-urban area of Uyo Metropolis, Nigeria

**DOI:** 10.11604/pamj.2017.28.303.14396

**Published:** 2017-12-08

**Authors:** Frances Sam Okpokowuruk, Mkpouto Udeme Akpan, Enobong Emmanuel Ikpeme

**Affiliations:** 1 Department of Paediatrics, University of Uyo Teaching Hospital, Uyo, Akwa Ibom State, Nigeria

**Keywords:** Hypertension, prevalence, children, age, gender, BMI, waist circumference socioeconomic status, family history

## Abstract

**Introduction:**

In the past, Hypertension in childhood was not considered a problem but in the last few decades, it has gradually become a source of concern especially as children are known to maintain their blood pressures into adulthood. Therefore, hypertensive children are at risk of developing cardiovascular complications earlier in adulthood. In our own environment, the prevalence of hypertension in children is undocumented, hence the purpose of this study.

**Methods:**

Two hundred children aged between 3-17 years were recruited into this study from two public schools-one primary, one secondary in a semi urban community in Uyo metropolis. The blood pressure of respondents was measured in accordance with the technique described by the 4^th^ Task Force on Blood Pressure Control in Children. The height and weight of all eligible subjects was measured using a stadiometer and a calibrated scale respectively. Body Mass Index (BMI) was assessed for each subject and World Health Organization (WHO) charts of BMI for age and sex were used as reference standards. Waist circumference was measured according to the technique described in the National Health and Nutrition Examination Survey.

**Results:**

The prevalence of hypertension and prehypertension was found to be 3.5% and 2.5% respectively in this study. Only age (OR = 1.74, p = 0.005, 95%CI = 1.186-2.566), BMI (OR = 1.54, p = <0.001, 95% CI = 1.249-1.913) and waist circumference (OR = 1.16, p = 0.002, 95%CI = 1.056-1.271) were found to significantly predict the development of high blood pressure.

**Conclusion:**

The prevalence of hypertension and prehypertension in this study was found to be low. Hypertension/prehypertension was more likely to develop with increasing age, BMI and waist circumference.

## Introduction

Hypertension is fast becoming a source of growing concern in children in developing countries [1]. Children with elevated blood pressures (BP) tend to maintain those same levels of blood pressure into adulthood, therefore, early detection is essential to minimize complications later in life [2]. In children, hypertension is said to be present when the systolic and diastolic blood pressure is greater than the 95^th^ percentile for the child's age, sex and height on three or more occasions [3]. The Fourth Report of the Task Force on Blood Pressure Control in Children commissioned by the National Heart, Lung and Blood Institute (NHLBI) has also introduced a new category of elevated blood pressure called prehypertension. This condition is diagnosed when a child´s average BP exceeds the 90^th^ percentile but is less than the 95^th^ percentile for age, sex and height. Any adolescent whose BP is greater than 120/80 mm Hg is also given this diagnosis, even if their reading is less than the 90^th^percentile [3]. Worldwide, the prevalence of hypertension in children ranges between 1-5% with a significant proportion of them being underdiagnosed [4]. In Nigeria, various studies done by Ujunwa et al. [5], Ejike et al. [6] and Okoh et al. [7] have also demonstrated similar prevalence rates of 5.4%, 4.6% and 4.7% respectively. In south south Nigeria, most of the studies on hypertension in children have been done mainly in Port Harcourt with none in Uyo, Akwa Ibom state [7, 8]. Since hypertension is a major risk factor for cardiovascular disease and there is evidence that childhood hypertension can predispose to hypertension in adulthood [9], this study is timely as it will help to add to the body of evidence on childhood hypertension and help bring about interventions to limit this non-communicable disease in our environment. This study was therefore undertaken to assess the prevalence of hypertension and prehypertension among children in Uyo and to determine the relationship, if any, between childhood hypertension and certain factors such as age, gender, socioeconomic status and family history of hypertension.

## Methods

This cross-sectional study was conducted in Etoi which is located to the north of Uyo metropolis, Akwa Ibom State, Nigeria. Etoi is one of the four clans that make up Uyo local government area and consists of twenty-two villages. Mbiabong is one of the villages in Etoi and consists of four communities. The population is predominantly Ibibio/Annang speaking. However, other ethnic groups such as Igbos, Yorubas and Hausas also reside there. A simple random sampling method was used to select Mbiabong community. Mbiabong has three primary schools and one secondary school. Consequently, one primary school was selected by random sampling from the three primary schools while the only secondary school was selected. Two hundred children aged between three and seventeen years irrespective of their ethnic group were recruited into the study, one hundred from each school. In each school, a total of seventeen students were selected randomly by a simple random sampling (balloting) technique from each class arm i.e. classes 1-6 for the primary school and junior (JS 1-3) and senior secondary school classes (SS1-SS3) for the secondary school. Sample size was calculated using the formula for calculating sample size for a cross sectional study [10] where z is the standard score corresponding to a confidence level of 1.96, d corresponds to a precision of 0.07 and p corresponds to a prevalence rate of hypertension of 4.7% [7].

$$\text{N}=\frac{{\text{z}}^{2}\text{pq}}{{\text{d}}^{2}}$$

Where: N = minimum sample size Z = 1.96 d = Total width of the expected confidence interval set at 0.07 p = prevalence from similar study (4.7%) [7] q = 1-p i.e. N = 1.96x1.96x0.47x0.53 = 0.95694256/0.0049 = 195 [200].

Details of the study objectives, procedures and potential benefits were explained to the study respondents during the initial visits and consent was sought and obtained from both respondents and their parents. Personal data such as age, gender and health condition (both previous and current) of the respondents was obtained using a structured questionnaire. Subjects were also classified into five social classes based on the Oyedeji socioeconomic classification scale [11]. This scale takes into cognizance both parents'/guardian's occupation and educational levels on a score of 1-5. The mean of the total of the four scores i.e. father's occupation + father's educational level + mother's occupation + mother's educational level divided by 4 to the nearest whole number gives the social class. Social class 1 is the highest class while social class 5 is the lowest but for the purposes of this study, social classes 1, 2 and 3 are classified as high socioeconomic class while classes 4 and 5 are classified as low socioeconomic class. This is as outlined below: Table 1. Family history of hypertension was also sought and obtained with a subject being classified as having a positive family history of hypertension only if the disease is present in a first-degree relative. The height and weight of all eligible subjects was measured using a stadiometer and a calibrated scale respectively recorded to one decimal place. Body Mass Index (BMI) was assessed for each subject and World Health Organization (WHO) charts of BMI for age and sex were used as reference standards. Respondents with BMI above the 95^th^ percentile were considered obese while those with BMI between the 85^th^ and 95^th^ percentile were considered overweight. Waist circumference was measured according to the technique described in the National Health and Nutrition Examination Survey (Anthropometry procedures manual) [12]. The Blood pressure was measured using the mercury gravity sphygmomanometer with an appropriate sized cuff for age being used. The subject was placed in a sitting position with the right upper arm placed on a table after the subject had been allowed to rest for at least 15 minutes before the procedure. The technique of BP measurement adopted was as described by the 4^th^ Task Force on Blood Pressure Control in Children [3].

**Table 1:** Oyediji socioeconomic classification scale

Score	Occupation	Educational level	Socioeconomic class (SEC)
1	Senior public servants, professionals, managers, Large scale traders, businessmen and contractors.	University graduates or equivalent	1-High SEC
2	Intermediate grade public servants and senior school teachers.	School certificate (ordinary level GCE) holders who have teaching or other professional training.	2-High SEC
3	Junior school teachers, drivers, artisans.	School certificate holders or grade II teachers certificate holders or equivalent.	3-High SEC
4	Petty traders, labourers, messengers and similar grades.	Modern 3 and primary 6 certificates.	4-Low SEC
5	Unemployed, students, full time housewives and subsistence farmers.	Those who can either just read and write or are illiterate.	5-Low SEC

The first Korotkoff sound was taken as the systolic pressure while the fifth Korotkoff sound was taken as the diastolic pressure. The blood pressure measurement was carried out by the three co-investigators separately on each subject with the average of the three readings taken as the blood pressure of the subject. Hypertension was diagnosed if blood pressure either systolic, diastolic or both was more than the 95^th^ percentile for age, sex and height while pre-hypertension was diagnosed if blood pressure ranged between the 90^th^ and 95^th^percentiles. Those with a diagnosis of pre-hypertension and hypertension had two subsequent consecutive blood pressure measurements at two weekly intervals. Data was analysed using the STATA statistical software version 10. Frequency tables were constructed for categorical variables while means and standard deviations were calculated for continuous variables. Ordinal logistic regression was carried out to identify factors significantly associated with hypertension and prehypertension. P-value < 0.05 was taken as the level of statistical significance. During the data analysis, no respondents in social classes 4 and 5 had prehypertension and there was no value for the RRR and 95% CI. For this reason, the social classes were re-categorized into 2 groups: groups 1-3 as high and groups 4-5 as low. Consent was sought from the parents while assent was obtained from the respondents as earlier stated and ethical approval was obtained from the University of Uyo Teaching Hospital ethics committee. Permission to conduct the study in the schools was also obtained from the state Ministry of Education.

## Results

Table 2 shows the sociodemographic and clinical characteristics of the study subjects. More than half (56%) of the respondents are between 13-17 years. The majority (64%) are females. Almost half (44%) belonged to social class 3. A greater proportion (81.5%) had a negative family history of hypertension with a normal BMI (95%). Table 3 shows the frequency of occurrence of hypertension and prehypertension with the prevalence of pre-hypertension and hypertension being 2.5% and 3.5% respectively. Table 4 shows the mean age, weight, height and waist circumference of respondents which was 12.44 years, 37.44Kg, 144.07cm and 61.738cm respectively. The mean systolic and diastolic blood pressures of respondents was 103.66 ± 15.63 and 64.87±11.19 mmHg respectively. Table 5 shows the factors which are associated with hypertension and prehypertension. Age (OR = 1.74, p = 0.005, 95%CI = 1.186-2.566), BMI (OR = 1.54, p = < 0.001, 95% CI = 1.249-1.913) and waist circumference (OR = 1.16, p = 0.002, 95%CI = 1.056-1.271) were significantly associated with developing hypertension when compared to being prehypertensive or normotensive. Gender, social class of respondents and family history are not associated with respondents' chances of being hypertensive compared to normotensive or prehypertensive in this study.

**Table 2:** Socio-demographic and clinical characteristics of respondents

Characteristics	Frequency	Percent
**Age Group (in years)**		
3-7	23	11.5
8-12	65	32.5
13-17	112	56.0
**Gender**		
Female	128	64.0
Male	72	36.0
**Social Class**		
1	13	6.5
2	38	19.0
3	88	44.0
4	55	27.5
5	6	3.0
**Family History**		
Negative	163	81.5
Positive	16	8.0
Unknown	21	10.5
**Body Mass Index**		
Normal	190	95.0
Low	9	4.5
High	1	0.5

**Table 3:** Frequency of Hypertension / prehypertension among respondents

Characteristic	Frequency	Percent
Prehypertension	5	2.5
Hypertension	7	3.5
Normotension	188	94.0

**Table 4:** Mean of blood pressure and anthropometric measurements of respondents

Anthropometric Characteristics	Mean+ standard deviation
Age	12.44±3.59
Weight (Kg)	37.34±12.87
Height (meters)	144.07 ±17.30
Waist circumference (cm)	62.73±7.38
SBP (mmHg)	103.66±15,63
DBP (mmHg)	64.87±11.19

**Table 5:** Factors associated with prehypertension and hypertension using ordinal logistic regression

Factor	Hypertension		
	OR	P value	95% Confidence Interval
**Gender**			0.151-2.207
Female	1		
Male	0.58	0.423	
Age	1.74	0.005	1.186-2.566
BMI	1.54	< 0.001	1.249-1.913
Waist circumference	1.16	0.002	1.056-1.271
^+^**Social class**			
High	1		0.094-2.102
Low	0.45	0.308	
**Family History**			
Negative	1		0.130-8.956
Positive	1.08	0.942	

+ High social class are classes 1-3; Low social class are classes 4-5

## Discussion

The prevalence rate for hypertension in this study was 3.5% and is similar to prevalence rates in early studies done by Einterz et al in Vandeikya (Benue State) who reported a rate of 3.3% [13] and Ayoola in Ibadan 3.3% [14]. More recent studies carried out by Bugaje et al in Zaria found a prevalence rate of 3.7% [15], Okoh et al., Okpere et al. and Also et al. in Port Harcourt and Kano respectively, also show similar prevalence rates ranging between 3-5% [7, 8, 16]. This suggests that the prevalence of hypertension in Nigerian children has remained remarkably constant over the last three decades. This trend has been corroborated by Ejike [17], who did a meta-analysis of data from studies in Nigeria spanning the past four decades and found that the prevalence rate for hypertension was 5.1% (for studies that used the JNC definition for hypertension) with only a slight decline over time. A meta-analysis of studies done by Monyeki and Kemper [18] found high prevalence rates of hypertension ranging between 7.5%-22.3% in children in Southern Africa when compared to the index study. In other parts of Africa such as Ghana in West Africa and in Tunisia in North Africa, prevalence rates of 6% and 9.6% respectively were obtained by Addo et al [19] and Harrabi et al [20]. The trend in Central Africa appears to be similar to what obtains in West Africa generally and in particular, in the index study where Kidy et al [21] in Uganda had prevalence rates of 3.8% while Mbolla et al in Congo Brazzaville had 3.3% [22]. This variation in prevalence rates in different parts of Africa may be explained by the differences in methods of blood pressure measurement and what study population was involved with many of the studies being done in adolescents. Large cohort studies done in the United States showed prevalence rates of 3.2 and 3.6% respectively [23, 24] while studies done in Asia in Surat city (Western India) gave a higher prevalence rate of 6.5% [25] though with a much smaller sample size comparatively. Overall, the worldwide prevalence of hypertension in children has been estimated to be between 1-5% of which the index study falls within the range [4]. Elevated blood pressure in childhood as a precursor to the latter development of hypertension in adulthood of the affected children has been well documented [26]. Prehypertension as a subgroup was created to identify children at the greatest risk for developing hypertensive disease in the future. The prevalence rate for prehypertension obtained in this study was 2.5% which is much lower than rates obtained in Vandeikya and Enugu which ranged between 17.3-25% [5, 6]. Both studies were done mainly in adolescents and may reflect the trend of higher rates of prehypertension being seen in adolescents when compared to the younger age groups. Okoh and Alikor in Port Harcourt, Nigeria had a prevalence of prehypertension of 4.7% [7] but the age group studied were preadolescent which may explain their much lower prevalence rates. This same trend of higher rates in adolescents is noted in studies done in South Africa, India and America [27-29]. However, in the index study, both adolescents and preadolescents were included in the study which implies that the prevalence of prehypertension in our own environment is still quite low when compared to others.

Factors which were significantly associated with developing hypertension in this study were age, BMI and waist circumference. As age increased, there was a higher likelihood of a child becoming hypertensive and this is in keeping with findings from other studies [5, 13, 16, 30]. This can be explained by the observed trend of increasing blood pressure with increase in body size, weight and sexual maturity which tends to occur with increasing age [31-33]. BMI continues to demonstrate a significant positive correlation with hypertension as seen in this study. Several studies corroborate this finding: Oduwole et al. [34] in Lagos, Nigeria found that BMI in adolescents aged 14-17years was significantly associated with prehypertensive and hypertensive range systolic blood pressures in overweight and obese subjects. Salman, Kirk and Deboer found that among primary school children in Khartoum, children who were obese had a high relative risk for developing systolic hypertension when compared to normal weight children [30]. Several other studies have consistently demonstrated this finding and in addition, found that waist circumference is also significantly associated with the development of hypertension in children as was seen in this study [35, 36]. In the index study, gender was not found to be significantly associated with developing hypertension in childhood. A meta-analysis of 21 studies done across Nigeria by Ejike [17] showed that there was no significant difference in the prevalence of hypertension between the sexes thereby corroborating the findings of this study. Socioeconomic status was not found to play a significant contributory role in the development of hypertension in this study although some studies show an association between low parental socioeconomic status and hypertension [37, 38]. Ansa et al. [39] have proposed that environmental and economic stressors in the low socioeconomic group may act as contributory factors to the development of hypertension. The relationship between a positive family history of hypertension and the presence of hypertension in children has been demonstrated by Okoh and Alikor [7] in Portharcourt and Mijinyawa et al in Kano [40] though in the index study, family history did not demonstrate any significant association with hypertension/prehypertension. This finding may be explained by the fact that many of the study participants were unaware of their family history of hypertension and thus responded to the contrary. The finding in this study is also corroborated by Buch et al in India [25] who did not find any significant relationship between family history of hypertension and hypertension in the offspring. A limitation of this study was its relatively small sample size compared to other large cohort studies done elsewhere though the prevalence rates obtained in this study were similar to those in other studies [22, 23]. Thus, the prevalence rates obtained in this study may not be a true indication of actual prevalence rates thereby necessitating a larger study in the future to help to clarify this.

## Conclusion

In conclusion, the prevalence of hypertension and prehypertension in our study populace appears to be low. Amongst the factors investigated in this study, only age, BMI and waist circumference were significantly associated with the development of hypertension and prehypertension. The low prevalence of hypertension and prehypertension in this study notwithstanding, with the increasing rate of urbanization prevalent in many Nigerian cities and the attendant changes in lifestyle, early health education is a prerequisite as an intervention to help in forestalling the morbidity/mortality from cardiovascular disease that has been observed in other environments.

### What is known about this topic

- Prevalence of hypertension in childhood varies from place to place;
- Hypertension in childhood can act as a precursor to hypertension in adulthood.

### What this study adds

- Prevalence of hypertension in the study populace is still low;
- Age, BMI and waist circumference were significantly associated with the development of hypertension and prehypertension in childhood.

## Competing interests

The authors declare no competing interests.
